# The Non-Operative Treatment of Varicose Veins of the Leg

**Published:** 1911-09

**Authors:** 


### The Non-Operative Treatment of Varicose Veins of the Leg

HACKETT (*Journal of the Michigan State Medical Society,* March 1911) thus describes the treatment advocated, which lies in the use of a boot composed of Unna's paste and ordinary gauze bandages. The paste is composed of the following: gelatin, white, 2 parts; zinc oxide, 2 parts; glycerin, 3 parts; aqua, 6 parts.

This is made over a water-bath, first completely dissolving the gelatin, then stirring in gradually the zinc oxide until it is thoroughly incorporated in the mixture, and last of all adding the glycerin.

Gelatin increases the plasticity of liquids with which it is incorporated, and it has unquestionable adhesive properties. Gelatin has been accused of causing tetanus, and in order to obviate this risk it should be thoroughly sterilised at a temperature of 120° C. An inch strip of adhesive plaster is placed around the limb below and above, to mark the margin of the boot. The hot paste is painted on with a brush all over the limb from the lower to the upper adhesive strip, and the gauze bandage two and one-half or three inches wide is applied from below; the gauze is wound snugly once around and overlapped and cut by the surgeon, while the assistant does the painting. Two or three layers of bandages are applied in this way and the boot allowed to dry. An ordinary stocking and shoe may be worn over the boot and the patient return to work.

If there is much excretion from an ulcer, a small window may be cut through the boot in a few days and the ulcer dressed.

The application of the paste in this manner is antiseptic, healing, and soothing to ulcers and varicose eczema, and gives support to the vessels better than a stocking or elastic bandage.

The boot may be worn from two weeks to two or three months, depending upon the condition present.

It is surprising how rapidly healthy granulations spring up from foul and even gangrenous ulcers under this treatment.—*Therapeutic Gazette.*

